# Benzo(a)pyrene Induced p53 Mediated Male Germ Cell Apoptosis: Synergistic Protective Effects of Curcumin and Resveratrol

**DOI:** 10.3389/fphar.2016.00245

**Published:** 2016-08-08

**Authors:** Bhaswati Banerjee, Supriya Chakraborty, Debidas Ghosh, Sanghamitra Raha, Parimal C. Sen, Kuladip Jana

**Affiliations:** ^1^Division of Molecular Medicine, Bose Institute, Calcutta Improvement Trust Scheme VIIMKolkata, India; ^2^Department of Bio-Medical Laboratory Science and Management, Vidyasagar UniversityMidnapore, India; ^3^Department of Biotechnology and Integrated Sciences, Visva BharatiShantiniketan, India

**Keywords:** B(a)P, germ cell, apoptosis, AhR, MAPK, p53

## Abstract

Benzo(a)pyrene (B(a)P) is an environmental toxicant that induces male germ cell apoptosis. Curcumin and resveratrol are phytochemicals with cytoprotective and anti-oxidative properties. At the same time resveratrol is also a natural Aryl hydrocarbon Receptor (AhR) antagonist. Our present study in isolated testicular germ cell population from adult male Wistar rats, highlighted the synergistic protective effect of curcumin and resveratrol against B(a)P induced p53 mediated germ cell apoptosis. Curcumin-resveratrol significantly prevented B(a)P induced decrease in sperm cell count and motility, as well as increased serum testosterone level. Curcumin-resveratrol co-treatment actively protected B(a)P induced testicular germ cell apoptosis. Curcumin-resveratrol co-treatment decreased the expression of pro-apoptotic proteins like cleaved caspase 3, 8 and 9, cleaved PARP, Apaf1, FasL, tBid. Curcumin-resveratrol co-treatment decreased Bax/Bcl2 ratio, mitochondria to cytosolic translocation of cytochrome c and activated the survival protein Akt. Curcumin-resveratrol decreased the expression of p53 dependent apoptotic genes like Fas, FasL, Bax, Bcl2, and Apaf1. B(a)P induced testicular reactive oxygen species (ROS) generation and oxidative stress were significantly ameliorated with curcumin and resveratrol. Curcumin-resveratrol co-treatment prevented B(a)P induced nuclear translocation of AhR and CYP1A1 (Cytochrome P4501A1) expression. The combinatorial treatment significantly inhibited B(a)P induced ERK 1/2, p38 MAPK and JNK 1/2 activation. B(a)P treatment increased the expression of p53 and its phosphorylation (p53 ser 15). Curcumin-resveratrol co-treatment significantly decreased p53 level and its phosphorylation (p53 ser 15). The study concludes that curcumin-resveratrol synergistically modulated MAPKs and p53, prevented oxidative stress, regulated the expression of pro and anti-apoptotic proteins as well as the proteins involved in B(a)P metabolism thus protected germ cells from B(a)P induced apoptosis.

## Introduction

Seminiferous tubule of testis harbors large number of germ cells at different stages of development and maturation in the spermatogenic cycle. These spermatogenic cells give rise to mature spermatozoa which are released into the tubular lumen as functional sperms by the process of spermatogenesis. Spermatogenesis is a complex and dynamic process of proliferation, differentiation and transformation of spermatogonia into mature spermatozoa in three major stages, the mitotic stage, the meiotic stage and the maturation stage (Poccia, 1986). Each of these stages represents a key element in the spermatogenic process. Alterations occurring in any of them could lead to the production of abnormal spermatozoa and reduce proliferation of spermatozoa. Thus, the understanding of processes related to spermatogenesis is critical for the assessment of male reproductive health. Maintenance of healthy germ cell population is crucial for the production of active mature sperm pool. Benzo(a)pyrene [B(a)P] is an environmental toxicant, results from the incomplete combustion of organic fuels. Previous studies have reported that B(a)P exposure leads to deleterious effects on male reproductive health (Ramesh et al., 2004; Mohamed et al., 2010; Chung et al., 2011). To date, limited studies have been conducted to ascertain the impact of B(a)P exposure on spermtogonial germ cells. Our previous studies have suggested that B(a)P induced oxidative stress, DNA damage and apoptosis in testis (Banerjee et al., 2016). Oxidative stress and DNA damage modulate various cellular processes that result in the induction of apoptosis. Apoptosis, the programmed cell death, is associated with the maintenance of proper testicular homeostasis, yet excess cell death can result in defective spermatogenesis leading to infertility (Mishra and Shaha, 2005). The signaling events leading to germ cell apoptosis can be divided into two major pathways involving either mitochondria (intrinsic) or death receptors (extrinsic). The studies related to the germ cell apoptosis by environmental toxicants demonstrated the involvement of both the mitochondria dependent; intrinsic and death receptor dependent; extrinsic apoptotic pathways. The intrinsic pathway is characterized by Bax translocation, cytochrome c release from mitochondria and activation of the initiator caspase 9 and the executioner caspases 3,6 and PARP cleavage. Members of the Bcl2 family proteins play a major role in governing this mitochondrial dependent pathway. Bcl2 family proteins have been grouped into three classes (Youle and Strasser, 2008). One class inhibits apoptosis (Bcl2, Bcl-XL, Bcl-W, MCL1, Bcl-B and A1), whereas the second class promotes apoptosis (Bax, Bak and Bok). A third divergent class of BH3-only proteins (Bad, Bik, Bid, Hrk, Bim, BMF, NOXA and PUMA) can bind and regulate the anti-apoptotic Bcl2 proteins to promote apoptosis (Labi et al., 2006). The pro-apoptotic family members like Bax and Bak induce permeabilization of the outer mitochondrial membrane that leads to the release of cytochrome c. Cytochrome c activates activator caspase 9 which further activates executioner caspases.

Extrinsic pathway of apoptosis is involved with Fas/Fas ligand (Fas/FasL) system. Fas-FasL is considered as an important pathway of transcription dependent apoptosis. FasL binding to Fas induces trimerization of Fas receptors, which recruits Fas Associated Death Domain (FADD). This Fas/FADD complex activates initiator caspase 8 or 10. This in turn cleaves and activates executioner caspases. Cross talk between these pathways occur at multiple levels (Sinha Hikim and Swerdloff, 1999).

Curcumin (diferuoylmethane) is a naturally occurring plant polyphenol present in ancient Indian spice Turmeric, with various beneficial properties. It is known to possess anti-oxidative, anti- inflammatory activity (Aggarwal et al., 2007) as well as anti-tumoric property (Shishodia et al., 2007). It was reported that curcumin inhibits tumor cell proliferation and protects normal cells from environmental toxicant induced apoptosis (Aggarwal et al., 2007). Few reports stated that curcumin prevented testicular germ cell apoptosis from various stressful conditions (Cort et al., 2013; Kanter et al., 2013). Natural phytochemicals, those are aryl hydrocarbon receptor (AhR) antagonists may have potential defensive mechanism against B(a)P induced cellular toxicity. Resveratrol (3,5,4′- trihydroxystilbene), a polyphenolic compound which is present in several plants and fruits especially in grapes, is a competitive antagonist for the AhR (Casper et al., 1999). Studies have shown several potential health benefits of resveratrol devoid of any toxicity (Pervaiz, 2003). Resveratrol is also considered as a potent antioxidant and it increases antioxidant status and inhibits ROS formation in many cells (Pervaiz, 2003). Resveratrol protects many normal cells from DNA damage and apoptosis by modulating the anti- and pro-apoptotic mediators as well as shows anti-tumoric activity (Pervaiz, 2003; Chakraborty et al., 2008). Resveratrol prevents B(a)P induced sperm cell damage and apoptosis (Revel et al., 2001). Recently we found that resveratrol prevented B(a)P induced BPDE- DNA adduct formation and steroidogenic dysfunction in testis (Banerjee et al., 2016). So, the possible protective role of naturally occurring phytochemicals against B(a)P induced testicular toxicity needs immediate consideration.

We demonstrated the combinatorial protective effects of dietary curcumin and resveratrol on B(a)P induced testicular germ cell apoptosis. The precise biochemical and cellular mechanisms involved in cyto-protection and anti-apoptotic effect of curcumin and resveratrol in combination after B(a)P exposure are relatively unknown. This study highlights the involvement of p53 and stress activated protein kinases in the protective effect of the aforesaid phytochemicals against B(a)P induced male germ cell apoptosis.

## Materials and Methods

### Materials

B(a)P, 1-Chloro-2,4-dinitrobenzene (CDNB), 5,50-dithiobis (2-nitrobenzoic acid) [DTNB], reduced nicotinamide adenine dinucleotide (NADH), oxidized glutathione (GSSG), phenazinemethosulphate (PMT), reduced glutathione (GSH), thiobarbituric acid (TBA), PCI (Phenol Chloroform Isoamylalcohol 25:24:1), curcumin and resveratrol were purchased from Sigma Chemical Company, St. Louis, MO, USA. TRIZOL reagent was purchased from Invitrogen (Carlsbad, CA, USA). Verso cDNA synthesis kit was purchased from Thermo Fisher (Waltham, MA, USA). In SITU Death Detection Kit, Fluorescien was purchased from Roche (Indianapolis, IN, USA). Antibodies for AhR, Apaf1, FasL, ERK1/2 and β Actin were purchased from Santacruz Biotechnology (Santacruz, CA, USA). Antibodies for CYP1A1, p38MAPK, PARP, Histone H3, tBid and VDAC were procured from Abcam (Cambridge, MA, USA); Bax, Bcl2, Cytochrome c, Caspase-9,8 3, JNK1/2, Akt, p53, phospho p53 (ser 15) were purchased from Cell Signaling Technology (Beverly, MA, USA). All the chemicals and reagents were analytical grade.

### Methods

#### Animals and Drug Treatment

Adult male Wistar rats (8 weeks of age; 150–200 g body weight) were housed in a temperature-controlled (21 ± 2°C) animal room at a constant 12/12-h light/dark cycle, with free access to food and water. All procedures were performed in accordance with the protocols approved by the Institutional Animal Ethical Committee (IAEC/BI/08/2012). Animals were segregated equally (*n* = 10) and randomly into seven treatment groups. Animals in Group I (Control) served as normal controls and were gavaged the same volume (0.2 ml) of vehicle (Corn oil) for 60 days. Animals in Group II (Cur) were gavaged curcumin orally at a dose of 50 mg/kg body weight for 60 days (Garg et al., 2008). Animals in Group III (Res) received resveratrol at the dose of 50 mg/kg of body weight daily through oral gavage for 60 days. Animals in Group IV (Res) received daily through oral gavage for 60 days. Group IV (B(a)P) animals received B(a)P at the dose of 5 mg/kg of body weight daily through oral gavage for 60 days. Animals in Group V (B(a)P+Cur) received curcumin (50 mg/kg) along with B(a)P (5mg/kg) for 60 days. Animals in Group VI (B(a)P+Res) were gavaged B(a)P (5mg/kg) along with resveratrol (50mg/kg of body weight) for 60 days. Animals in Group VII (B(a)P+Cur+Res) were given a combined treatment of curcumin (50 mg/kg) and resveratrol (50 mg/kg) along with B(a)P (5mg/kg) for 60 days. We have selected the single dose for B(a)P, curcumin and resveratrol from our previous studies, as these have been the most effective doses under our experimental conditions (Supplementary Figures S1 and S2). Animals receiving only curcumin, resveratrol and in combination did not show any adverse effect (Supplementary Figure S2). The study was performed strictly accordance with the protocols of the National Institute of Health guidelines for the Care and Use of Laboratory Animals (NIH publication No. 85 –23 revised 1985: US Department of Health, Education and Welfare, Bethesda, MD, USA). The experimental outline also met the National Guidelines on the Proper Care and Use of Animals in Laboratory Research (Indian Science Academy, New Delhi, India) and the protocol was approved by the Institutional Animal Ethics Committee (IAEC) of Bose Institute, Kolkata, India. The animal breeding and experimental facility are registered with the Committee for the Purpose of Control and Supervision of Experiments on Animals (CPCSEA), Ministry of Environment and Forest and Climate Change, Government of India. Euthanasia was performed by decapitation under sodium pentobarbital anesthesia.

#### Epididymal Sperm Count and Motility and Relative Organ Weight

Sperm cells were collected from the cauda epididymis of each rat by flushing with same volume (1 ml) of suspension medium containing 140 mM NaCl, 0.3 mM KCl, 0.8 mM Na_2_HPO_4_, 0.2mM KH_2_PO_4_ and 1.5 mM D-glucose (pH 7.3). A fraction of suspension (200 μl) was mixed with an equal volume of 1% Trypan blue in the same medium, and numbers of sperms were counted in hemocytometer slide (Alvarez and Storey, 1984). Motility of spermatozoa was identified under a phase contrast microscope at ×400 magnification. An aliquot of the freshly extracted sperm preparation (1 × 10^6^ cells) was incubated with the above said medium for 15 min at room temperature before assessing the sperm motility with a hemocytometer. For the microscopic method of assay of sperm motility (expressed as %), all cells which showed some degree of motility (vibrating, progressive motility) was counted. All animals were weighed at the end of the treatment and the reproductive organs were weighed after their sacrifice. Experiments were repeated three times.

#### Histology Study

The formalin fixed testis from each animal groups were embedded in paraffin wax and 5 micron thick sections were cut from the blocks and stained with Hematoxylin-Eosin (H&E) and examined under light microscope at ×200 magnification.

#### Assay of Serum Testosterone Concentration

Serum Testosterone concentrations were determined in treated and control animals using Calbiotech ELISA kit using manufacturer’s instructions. The sensitivity of the assay is 0.075 ng/ml. The intra assay co-efficient of variation is 2.9% and inter assay coefficient of variation is 3.4%.

#### Germ Cell Isolation from Testis

A two-step enzymatic method with a few modifications (Ikeda et al., 1999) was performed for the isolation of germ cells from testis. The excised testis was rinsed in Hank’s balanced salt solution (HBSS) containing 5 mM glucose. The seminiferous tubule mass was incubated in HBSS containing 0.25 mg/ml collagenase for 15 min at 34°C with constant shaking. The dispersed tubules were washed with HBSS, which largely removed contamination from Leydig and blood cells. The isolated tubules were treated with trypsin (1.25 mg/mL) and DNaseI (50 μg/mL). The resultant crude cell suspension was filtered through organza, washed and re-suspended in DMEM-F12. The final viable cell population was about 95% and mostly contained maturing germ cells.

#### Viability Assay: Trypan Blue Dye Exclusion Test

Viability of the cells was determined by Trypan Blue dye exclusion test. The cell suspension was mixed with 0.125% Trypan Blue dye (wt/volume in sterile isotonic saline). The cells were viewed within 10 min with a light microscope at 40X and 50–100 cells counted with hemocytometer. Viable cells remained unstained, while the nuclei of non-viable cells were stained blue. The ratio of viable cells to the total number of cells counted was recorded as a percent viability for each sample. Calculations was made as follows, Cells/ml = the number of cells per quadrant equals 10^4^ cells/ml (50 cells per quadrant **=** 0.5 million cells/ml); Total cells: cells/ml × original volume; Cell viability (%) = total viable cells (unstained)/total cells (stained and unstained) × 100.

#### Annexin V-PI Staining

Cell death was determined by staining the cells with Propidium Iodide and AnnexinV-FITC (BD Pharmingen, San Jose, CA, USA) and analyzed on flow cytometer, (FACS Caliber, Beckton Dickinson, San Diego, CA, USA) equipped with 488 nm Argon laser light source, using Cell Quest Software (BD Biosciences, San Jose, CA, USA). 10,000 events were acquired for analysis. AnnexinV-FITC positive cells were denoted as apoptotic cells.

#### Western Blotting

Germ cells were lysed in RIPA buffer to prepare whole cell lysate. Nuclear, mitochondrial and cytosolic fractions were prepared using standard protocols (Li et al., 2003; Liang et al., 2013). All the buffers were supplemented with protease and phosphatase inhibitor cocktails. Equal amount of protein (50 μg) of each sample was resolved on 10% SDS–PAGE and electrophoretically transferred to PVDF membranes. The membrane was blocked with 5% non-fat milk in TBST for 1 h and then incubated with specific primary antibodies for CYP1A1 (Abcam, USA), AhR, Apaf1, phospho and complete ERK1/2, FasL (Santacruz, USA), phospho and complete p38 MAPK, tBid (Abcam), Bax, Bcl2, cytochrome c, caspase 9,8,3, phospho and complete JNK1/2, phospho and complete p53 (Cell signaling), and PARP (Abcam) at 4°C overnight and subsequently exposed to HRP conjugated secondary antibodies. The bands were visualized with ECL detection. β Actin (Santacruz, USA) was used as the whole cell and cytosolic fraction loading control, VDAC (Abcam, USA) was used as the loading control for the mitochondrial fraction and Histone H3 (Abcam, USA) was used as the loading control for the nuclear fraction.

#### Reverse Transcriptase PCR

mRNA expression of apoptotic genes were estimated using semiquantitative reverse transcriptase PCR method. Briefly, total RNA from cells was extracted with TRIZOL reagent (Invitrogen, Carlsbad, CA, USA) and cDNA was synthesized using the Verso cDNA synthesis kit (Thermo, USA). cDNA was subjected to PCR (30 cycles) for CYP1A1, AhR, Apaf1, Fas, FasL, Bcl2, Bax and β Actin in 20 μL reaction mixture [10× PCR buffer, 2.5 mM dNTP (deoxyribonucleotide triphosphate), Taq-polymerase 1U, and forward and reverse primers]. The PCR products were resolved by 1% agarose gel electrophoresis and visualized using Ethidium bromide. The primer sequences are stated in the **Table 1**.

**Table 1:** Primer sequences used for reverse transcriptase PCR.

Primer	Forward (5′-3′)	Reverse (5′-3′)	Product size (bp)
Fas	AAATGAAAGCCAACTGCATCGAC	ATTGGACCCTCGCTGAGCAC	88
FasL	CACCAACCACAGCCTTAGAGTATCA	ACTCCAGAGATCAAAGCAGTTCCA	171
p53	AGATGTTCCGAGAGCTGAATG	ACAACTGACCGGATAGGATTTC	106
CYP1A1	CTGGTTCTGGATACCCAGCTG	CCTAGGGTTGGTTACCAGG	331
AhR	GGGATCGATTTCGAAGACATCAG	AACGCCTGGGAGCCTGGAATCTC	233
Bcl2	CATGCGACCTCTGTTTGATTC	GAATGTGTGTGTGTGTGTGTG	118
Bax	GGCAGACAGTGACCATCTTT	CCAAAGTGGACCTGAGGTTTAT	136
Apaf1	CAGTGCTTTCCTGTGCTATCT	GGTAGCCGTTCCTTCTTCTATG	205
β Actin	GGAGATTACTGCCCTGGCTCCTA	GACTCATCGTACTCCTGCTTGCTG	150

#### Measurements of ROS, Oxidative Stress and Antioxidant Status in Testis

Intracellular ROS production was estimated by using 2, 7-dichlorofluorescein diacetate (DCF-DA) as a probe according to the method of Kuo and Tang (1998). Briefly, 100 μl of testis homogenate from pooled testes was incubated with the assay media (20 mM tris-HCl, 130 mM KCl, 5 mM MgCl_2_, 20 mm NaH_2_PO_4_, 30 mM glucose and 5 μM DCF-DA) at 37°C for 15 min. The formation of DCF was measured at the excitation wavelength of 488 nm and emission wavelength of 510 nm for 10 min by using spectrofluorometer (HITACHI, Model No F7000) equipped with a FITC filter.

For oxidative stress measurement the testicular tissue was homogenized in ice-cold 0.1 M Tris-HCl buffer (pH7.4) and centrifuged at 10,000 × *g* at 4°C for 10 min and the supernatant was collected. The protein content of the preparation was determined. The supernatant was used for the following biochemical assays. All experiments were performed in triplicates under the same experimental conditions.

The level of lipid peroxidation was measured by colorimetric reaction with thiobarbituric acid (TBA) as described by the method of Devasagayam and Tarachand (1987). The absorbance of thiobarbituric acid reactive substance (TBARS) formed was measured at 532 nm and its concentration was calculated using the extinction coefficient of MDA which is 1.56 × 10^5^ M^-1^ cm^-1^. The MDA content of the sample was expressed as nano moles of MDA formed per milligram protein.

Activity measurement of Catalase was performed according to Beers and Sizer (1952), monitoring the decrease of H_2_O_2_ at 240 nm. One unit of Catalase activity is defined as the amount of enzyme, which reduces 1 μM of H_2_O_2_ per minute.

Reduced and oxidized glutathione levels were measured by the method of Hissin and Hilf (1976) using *o*-phthalaldehyde (OPT) as a fluorescent reagent. The method takes advantage of the reaction of GSH with OPT at pH 8 and of GSSG with OPT at pH 12. GSH can be complexed to *N*-ethylmaleimide to prevent interference of GSH with measurement of GSSG. The fluorescence was determined at excitation wavelength of 360 nm and emission wavelength of 460 nm. The result was represented as the amount of reduced GSH as well as the ratio of reduced GSH/GSSG.

The activity of glutathione peroxidase (GPx) was determined by the method of Rotruck et al. (1973). GPx activity was assayed using H_2_O_2_ and GSH as substrates. The absorption intensity was measured at 412 nm. One unit of enzyme activity was expressed as units per mg protein (1 U is the amount of enzyme that converts 1 μM GSH to GSSG in the presence of H_2_O_2_ per minute.

#### Terminal Deoxynucleotidyl Transferase Enzyme Mediated dUTP Nick End Labeling (TUNEL) Staining of Testis Tissue Section

TUNEL assay of paraffin embedded testis tissue sections were performed with IN SITU Death Detection Kit, (Fluorescien, Roche) according to the manufacturer’s instruction (Roche, Indianapolis, IN, USA). Cell nuclei were counterstained with DAPI. Sections were observed under confocal microscope (Leica SP8, Germany) at ×200 magnification.

#### DNA Ladder Assay of Germ Cells

Cells were lysed with RIPA buffer and genomic DNA was isolated with PCI (Phenol Chloroform Isoamyl alcohol 25:24:1) (Sigma Chemical Company, St. Louis, MO, USA). DNA was resolved in 1% agarose gel and visualized with Ethidium bromide.

#### Evaluation of Oral Bioavailability and Pharmacokinetics Study of Resveratrol

The oral bioavailability of resveratrol and its major metabolites resveratrol-3-*O*-glucuronide and resveratrol-3-*O*-sulfate was investigated in the presence and absence of curcumin. Resveratrol was gavaged at 50 mg/kg body weight and curcumin was administrated at 50 mg/kg body weight. Blood samples were taken regularly from each rat into heparinized tubes at 0, 0.25, 0.5, 1, 2, 4, 8, 12, and 24 h after oral administration of resveratrol and curcumin. The plasma was separated by centrifugation and stored at -80°C for HPLC analysis. The areas-under-the curve (AUC) for the plot of the plasma concentration over time was evaluated using linear trapezoidal method. Cmax and Tmax were directly obtained from the curves.

#### Statistical Analysis

The results were expressed as mean ± SEM. One-way ANOVA was followed by Dunnett multiple comparison test. The level of significance was set at (^∗∗∗^*P* < 0.001); (^∗∗^*P* ≤ 0.01–0.001); (^∗^*P* ≤ 0.01–0.05) in respect with the control. GraphPad Prim 5.0 software was used to statistically analyze the data.

## Results

### Curcumin and Resveratrol Effectively Prevent B(a)P Induced Alteration in Relative Organ Weight, Decrease in Sperm Cell Count, Motility, Serum Testosterone Level, and Changes in Testicular Histoarchitechture

**FIGURE 1:**
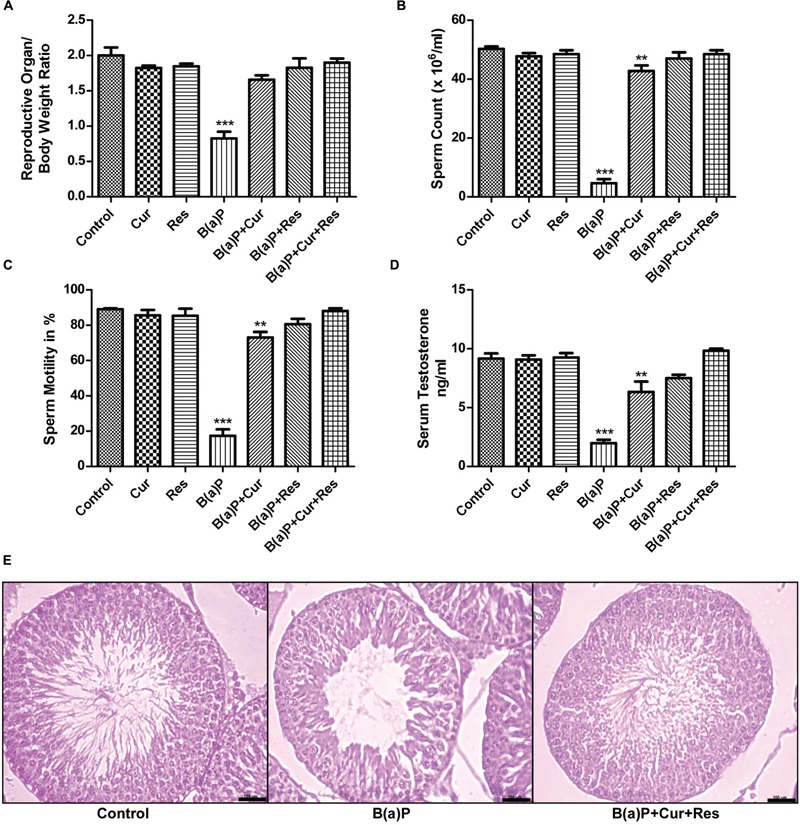
**Curcumin and resveratrol prevent B(a)P induced reproductive toxicity. (A)** Relative weight of reproductive organs in different study groups. Graphical representation of **(B)** sperm count (× 10^6^/ml) and **(C)** sperm motility (in %) and **(D)** serum testosterone level (ng/ml) in different study groups. The results were expressed as mean ± SEM. One-way ANOVA was followed by Dunnett multiple comparison test. The level of significance was set at (^∗∗∗^*P* < 0.001); (^∗∗^*P* ≤ 0.01–0.001); (^∗^*P* ≤ 0.01–0.05) in respect with the control. **(E)** H&E staining of paraffin embedded testis sections were visualized at ×200 magnification. Bar = 100 μm.

B(a)P induced significant damage to male reproductive system. We found that curcumin and resveratrol treatment prevented the B(a)P induced decrease in relative organ weight (reproductive organ/body weight ratio) (**Figure 1A**). B(a)P exposure resulted decrease in sperm count and motility. Our findings revealed that curcumin and resveratrol co-treatment significantly ameliorated the effect of B(a)P in sperm cells. In control animals the cauda epididymal sperm count was approximately 50 × 10^6^/ml whereas, B(a)P treatment brought down the sperm count to nearly 10 × 10^6^/ml. Curcumin and resveratrol co-treatment was able to restore the sperm count close to the normal (**Figure 1B**). Sperm motility is another major parameter critically affected by B(a)P. Our results indicated B(a)P mediated reduction in sperm motility was significantly restored by curcumin and resveratrol co-treatment (**Figure 1C**). Our findings also revealed that curcumin and resveratrol co-treatment was able to improve the B(a)P induced decrease in circulating testosterone concentration (**Figure 1D**). We have selected our dose of curcumin and resveratrol from dose dependent study (Supplementary Figure S2). H&E staining of rat testis showed that B(a)P caused significant degenerative changes. Furthermore curcumin-resveratrol co-treatment improved tubular sperm cell load, and prevented germ cell sloughing and tubular derangement (**Figure 1E**). All these findings together thus highlighted the role of curcumin and resveratrol against testicular protection from B(a)P induced toxicity.

### Curcumin and Resveratrol Effectively Protect B(a)P Induced Testicular Germ Cell Death and Apoptosis

**FIGURE 2:**
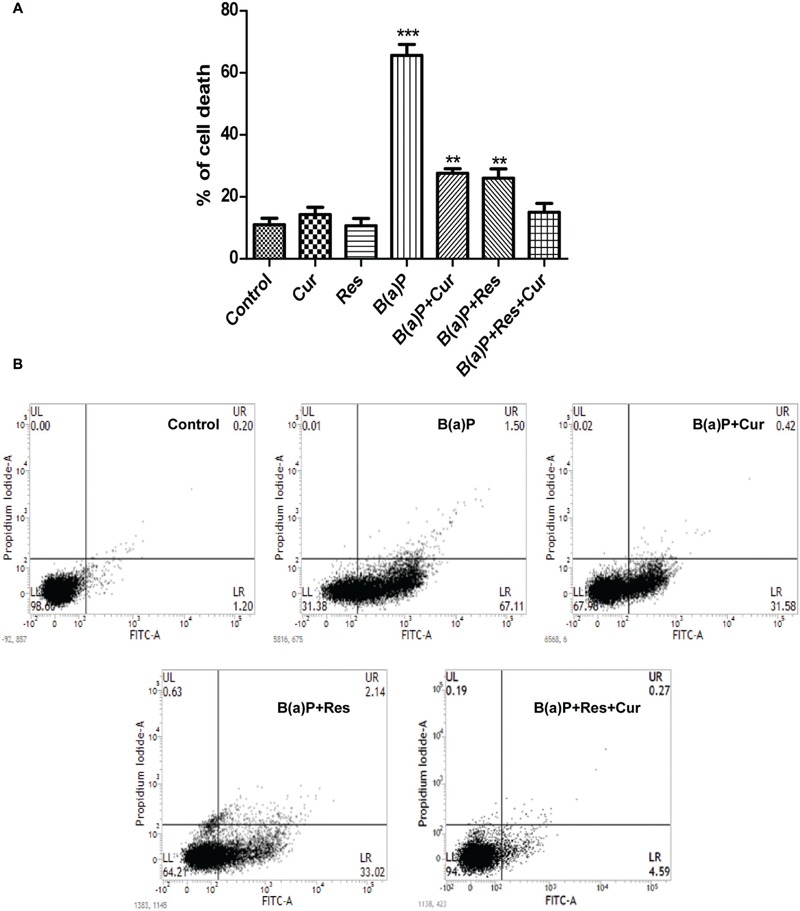
**Curcumin and resveratrol prevent B(a)P induced testicular germ cell death. (A)** Graphical representation of the percentage of cell death in isolated testicular germ cells in different animal groups, as determined by Trypan blue exclusion assay. **(B)** Germ cell death was determined by Annexin V-FITC staining assay. The results were expressed as mean ± SEM. One-way ANOVA was followed by Dunnett multiple comparison test. The level of significance was set at (^∗∗∗^*P* < 0.001); (^∗∗^*P* ≤ 0.01–0.001); (^∗^*P* ≤ 0.01–0.05) in respect with the control.

B(a)P induces apoptosis in testicular cells (Jiang et al., 2013; Das et al., 2014; Michurina et al., 2014; Nie et al., 2014). Reports from our laboratory have shown that B(a)P induced apoptosis in testis (Banerjee et al., 2016). Curcumin is a well known anticancer agent and it can protect normal cells from environmental toxicant induced apoptosis (Aggarwal et al., 2007). Resveratrol is considered as a potent antioxidant and natural AhR antagonist. Resveratrol also shows anti-apoptotic activity in different testicular cells (Banerjee et al., 2016). Germ cells undergo spermatogenic cycle and produces mature sperm cells. Our results showed that curcumin and resveratrol co-treatment significantly inhibited B(a)P induced germ cell death (**Figure 2A**). Cell viability assay was performed in isolated testicular germ cells. Approximately 70% germ cell death was detected in B(a)P treated animals. Curcumin and resveratrol individual treatment showed partial protection against B(a)P induced cell death. Whereas, curcumin and resveratrol co-treatment significantly decreased cell death (∼10%), which was nearly similar to the control condition. This result provided us the idea for the combinatorial cyto-protective activity of curcumin and resveratrol against B(a)P. Dose-dependent study of curcumin and resveratrol indicated that the combinatorial dose of curcumin (50 mg/kg) and resveratrol (50 mg/kg) were most effective against B(a)P (5 mg/kg) (Supplementary Figure S1). But increasing the dose of curcumin and resveratrol (100 mg) did not show any further improvement in their functions. We performed the Annexin V-PI assay with isolated germ cells to ascertain the nature of cell death (**Figure 2B**). The result reconfirmed our previous findings that B(a)P induced germ cell apoptosis (Banerjee et al., 2016). Curcumin and resveratrol co-treatment protected germ cells from B(a)P induced apoptosis. Apoptotic FITC positive cell count decreased to 5% in curcumin-resveratrol co-treated group from B(a)P treated group, where the apoptotic cell population count was approximately 67%. These findings pushed us toward the mechanistic study of B(a)P induced germ cell apoptosis and subsequent protection with curcumin and resveratrol.

### Curcumin and Resveratrol Prevent B(a)P Induced Caspase Dependent Testicular Germ Cell Apoptosis

**FIGURE 3:**
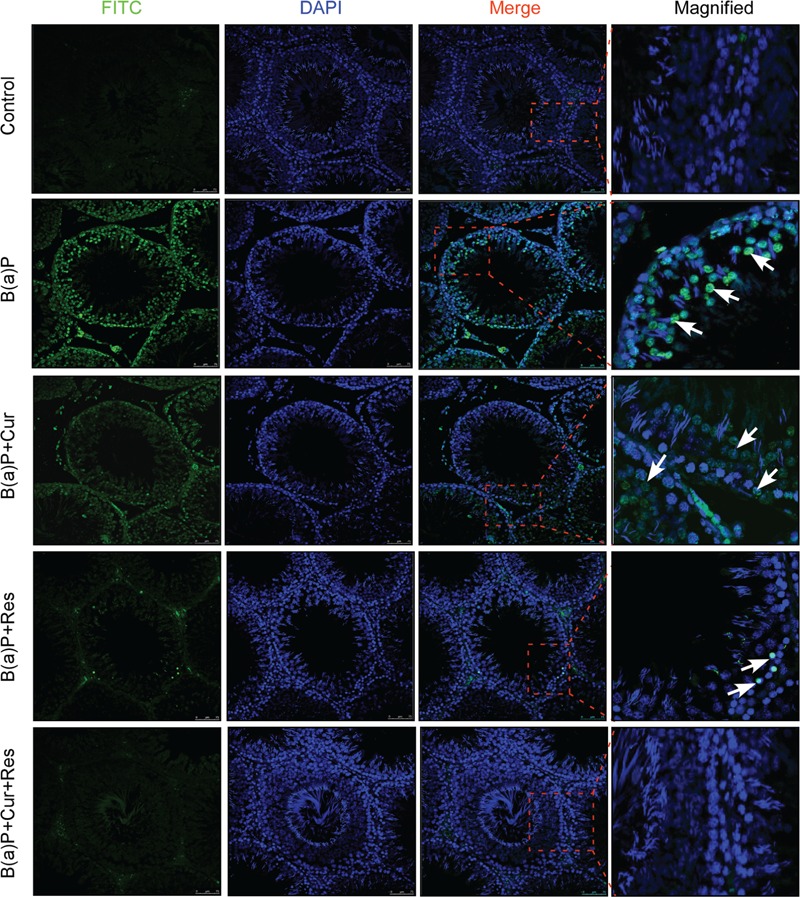
**Curcumin and resveratrol prevent B(a)P induced testicular apoptosis.** TUNEL staining of paraffin embedded testis sections at ×200 magnification. TUNEL-FITC positive apoptotic germ cells were stained green. Cell nuclei were counter stained with DAPI (blue). TUNEL positive spermatogonial cells were indicated with white arrow. Bar = 75 μm.

**FIGURE 4:**
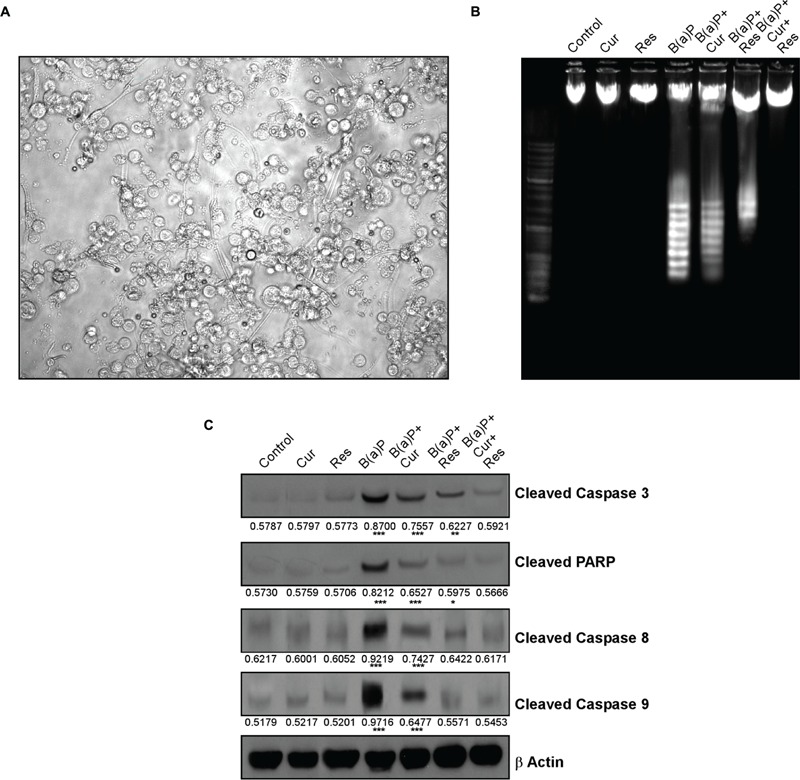
**Curcumin and resveratrol prevent B(a)P induced caspase dependent testicular germ cell apoptosis. (A)** Light microscopic image of isolated testicular germ cells at ×400 magnification. **(B)** DNA ladder assay of isolated germ cells. **(C)** Western blot analysis for cleaved caspase 3, cleaved PARP, cleaved Caspase 8 and cleaved Caspase 9. β Actin was used as the internal loading control. The numerical values indicated below the immunoblots are the densitometric analysis values expressing mean ± SEM of relative arbitrary units of the bands for three immunoblots were conducted with separate experiments in each group. One-way ANOVA was followed by Dunnett multiple comparison test. The level of significance was set at (^∗∗∗^*P* < 0.001); (^∗∗^*P* ≤ 0.01–0.001); (^∗^*P* ≤ 0.01–0.05) in respect with the control.

Germ cells comprise majority of the testicular cell population. They undergo several divisional steps to attain maturity. We performed TUNEL assay in the testis section of different experimental animal groups (**Figure 3**) to identify apoptotic germ cells in their *in situ* location. The predominant apoptotic cells after B(a)P treatment were spermatogonial cells. Curcumin-resveratrol co-treatment decreased the number of TUNEL positive cells (**Figure 3**). Testis comprises other different type of cells (Leydig, Sertoli cells). Our focus for the current study was germ cells. So, for the signaling study, we isolated the germ cell populations from testis (**Figure 4A**). DNA ladder formation is a significant marker for apoptosis. We performed DNA ladder assay from isolated germ cells. Results showed that B(a)P induced prominent DNA ladder and curcumin-resveratrol co-treatment significantly prevented DNA ladder formation (**Figure 4B**). The molecular mechanisms involving B(a)P induced cell death were delineated and we observed that B(a)P induced the activation of caspase 9, 8, and 3 and PARP (**Figure 4C**). It gave us the idea for the activation of both extrinsic and intrinsic apoptotic pathways in germ cells. Results indicated that curcumin alone could not significantly prevent B(a)P induced activation of caspase 9, 8, and 3 and PARP. Resveratrol alone prevented the activation of caspase 9 and 8. Combinatorial treatment of curcumin and resveratrol significantly prevented B(a)P induced activation of caspase 9, 8, and 3 and PARP. Thus curcumin and resveratrol together acted as the powerful effective players against B(a)P induced germ cell apoptosis.

### Curcumin and Resveratrol Co-treatment Prevents B(a)P Induced Activation of Intrinsic and Extrinsic Apoptotic Pathways in Germ Cells

**FIGURE 5:**
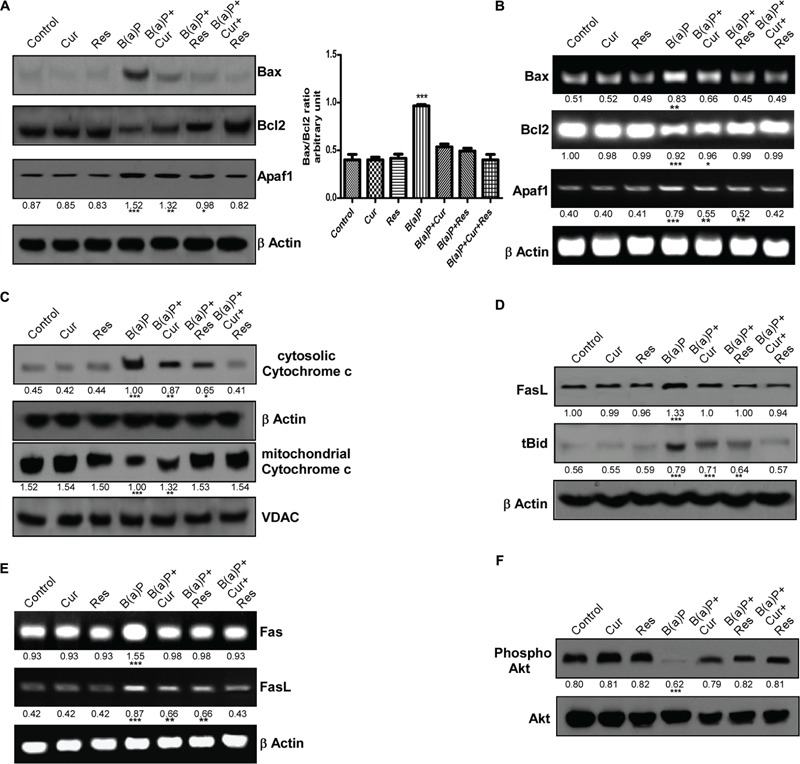
**Protective effect of curcumin and resveratrol against B(a)P induced testicular germ cell apoptosis. (A)** Western blot of Bax, Bcl2 and Apaf1. The corresponding histogram (right panel) showing Bax/Bcl2 ratio, expressing mean ± SEM of relative arbitrary units of the bands for three immunoblots were conducted with separate experiments in each group. One-way ANOVA was followed by Dunnett multiple comparison test. The level of significance was set at (^∗∗∗^*P* < 0.001); (^∗∗^*P* ≤ 0.01–0.001); (^∗^*P* ≤ 0.01–0.05) in comparison with the control. β Actin was used as the internal loading control. **(B)** RT-PCR analysis of Bax, Bcl2 and Apaf1 in different study groups. β Actin was used as the internal loading control. **(C)** Western blot of cytochrome c (cytosolic and mitochondrial fraction). β Actin was used as the internal loading control for whole cell and cytosolic fractions. VDAC was used as the loading control for mitochondrial fraction. **(D)** Western blot of FasL and tBid. β Actin was used as the internal loading control. **(E)** RT-PCR analysis of Fas and FasL in different study groups. β Actin was used as the internal loading control. **(F)** Western blot of phospho and total Akt. The numerical values indicated below the immunoblots and PCR bands are the densitometric analysis values expressing mean ± SEM of relative arbitrary units of the bands for three immunoblots, conducted with separate experiments in each group. One-way ANOVA was followed by Dunnett multiple comparison test. The level of significance was set at (^∗∗∗^*P* < 0.001); (^∗∗^*P* ≤ 0.01–0.001); (^∗^*P* ≤ 0.01–0.05) in respect with the control.

Our findings indicated B(a)P induced activation of intrinsic and extrinsic apoptotic pathways in testicular germ cells. Activation of both caspase 8 and 9 suggested that both ligand mediated and mitochondria mediated apoptosis were taking place. Here we have delineated the role of the molecular players associated with B(a)P induced germ cell apoptosis. Activation of intrinsic apoptotic pathway results in the up-regulation of pro-apoptotic Bax expression and the down-regulation of anti-apoptotic Bcl2 expression. This change of Bax/Bcl2 ratio disrupts the mitochondrial membrane potential that results in the cytosolic release of mitochondrial matrix protein cytochrome c. Cytochrome c forms apoptosome complex with another protein Apaf1. This complex activates the initiator caspase 9 that further activates the executioner caspases. Our results showed that B(a)P exposure significantly altered the cellular Bax/Bcl2 rheostat (**Figure 5A**, left and right panel). Curcumin and resveratrol individual treatment along with their co-treatment was able to reverse back the deleterious changes of Bax and Bcl2 ratio (**Figure 5A**, left and right panel). Curcumin-resveratrol co-treatment significantly decreased the transcriptional level of Bax and increased the transcriptional level of Bcl2 as similar to the control condition (**Figure 5B**). B(a)P treatment increased Apaf1 protein and mRNA expressions (**Figures 5A,B** respectively). Though curcumin alone did not show significant effect on Apaf1 expression but resveratrol treatment and its co-treatment with resveratrol brought back Apaf1 expression close to the control level (**Figures 5A,B**). Curcumin and resveratrol co-treatment resulted significant inhibition of B(a)P induced cytosolic translocation of cytochrome c (**Figure 5C**). Our results showed increased expression of active caspase 8 upon B(a)P exposure and its subsequent decrease with curcumin and resveratrol treatment. As caspase 8 is the initiator caspase for death receptor mediated apoptotic pathway, we examined the protein expression change of FasL (**Figure 5D**) as well as mRNA expression of Fas and FasL (**Figure 5E**). B(a)P exposure resulted in the increased FasL expression. Curcumin treatment alone was not significantly able to bring down the up-regulated expression of FasL. Curcumin and resveratrol co-treatment significantly decreased the B(a)P induced increased expression of FasL (**Figure 5D**). Bid is a Bcl2 family protein that forms the bridge between extrinsic and intrinsic apoptotic pathways. Caspase 8 activates Bid to tBid form. tBid translocates to mitochondrial membrane and facilitates cytochrome c release, thus activates the mitochondrial apoptotic pathway. Our results indicated that B(a)P exposure significantly increased tBid expression (**Figure 5D**). Whereas curcumin and resveratrol co-treatment significantly decreased tBid expression (**Figure 5D**). We further investigated the status of survival protein Akt in germ cells. We found that phospho Akt level was significantly decreased upon B(a)P treatment. Both curcumin and resveratrol treatment along with their co-treatment was able to recover the phospho Akt level significantly (**Figure 5F**).

### Curcumin and Resveratrol Co-treatment Protects the Testis from B(a)P Induced Oxidative Stress by Scavenging ROS and Improves the Testicular Anti-oxidant Status

**FIGURE 6:**
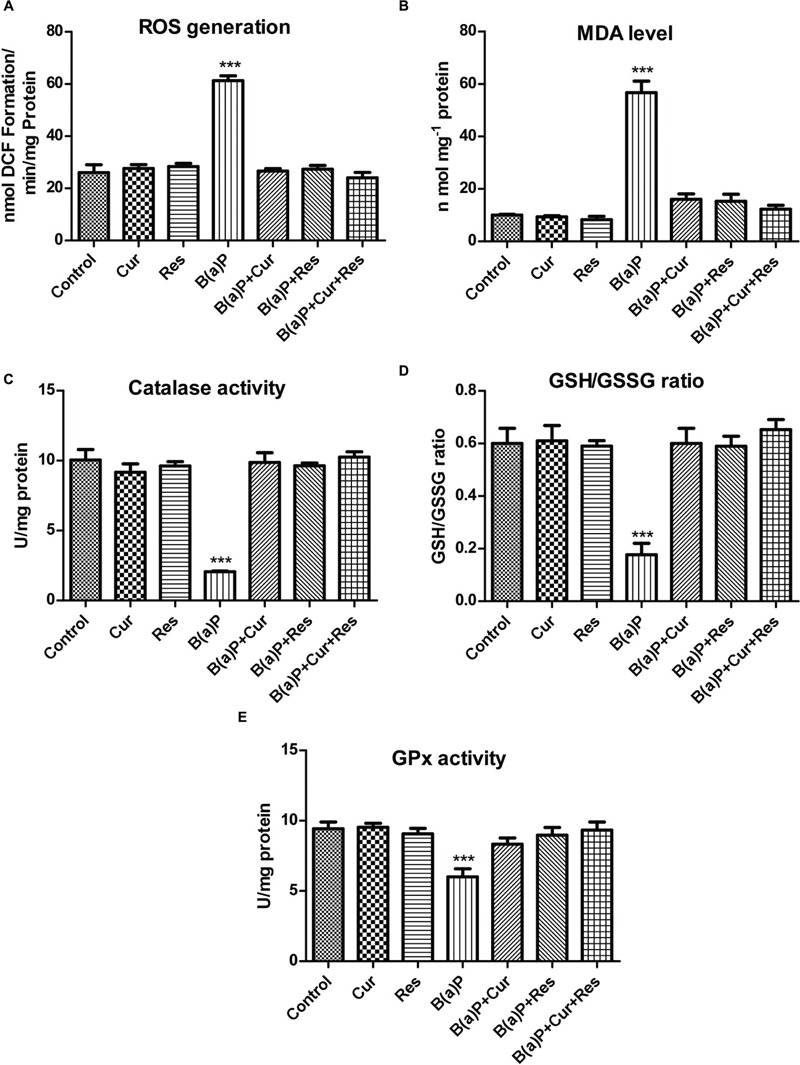
**Curcumin and resveratrol protects testicular germ cells from B(a)P induced ROS generation and oxidative stress. (A)** Graphical representation of ROS generation in testis. **(B)** Graphical representation of testicular MDA level (nmol/mg protein). **(C)** Graphical representation of activity of testicular Catalase (U/mg protein). **(D)** Graphical representation of testicular GSH/GSSG ratio. **(E)** Graphical representation of activity of testicular Glutathione Peroxidase (GPx) (U/mg protein). Each value represents mean ± SEM, *n* = 6. One-way ANOVA was followed by Dunnett multiple comparison test. The level of significance was set at (^∗∗∗^*P* < 0.001); (^∗∗^*P* ≤ 0.01–0.001); (^∗^*P* ≤ 0.01–0.05) as compared with the respective control.

We have found ROS mediated apoptosis by B(a)P (Banerjee et al., 2016). To study the role of curcumin and resveratrol against B(a)P induced oxidative stress, ROS generation, lipid peroxidation and antioxidant status in testicular tissue were checked (**Figure 6**). B(a)P increased testicular ROS generation while suppressed the anti-oxidative protection system in testis. Curcumin and resveratrol being natural antioxidant, we hypothesized their ROS scavenging activity against B(a)P. Our results indicated that curcumin and resveratrol either separately or in co-treatment significantly (^∗^*p* < 0.05) reduced testicular ROS generation (**Figure 6A**). Membrane lipid peroxidation is a major indicator of membrane lipid degradation, which results into apoptosis. Malondialdehyde (MDA) is the marker element of membrane lipid peroxidation. Curcumin, resveratrol and curcumin-resveratrol co-treatment with B(a)P significantly declined the MDA level in testicular cells (**Figure 6B**). B(a)P significantly declined the activity of another associated anti-oxidative enzyme catalase. Curcumin, resveratrol and curcumin-resveratrol co-treatment was able to restore the activity of catalase (**Figure 6C**). Glutathione being the major anti-oxidative enzyme protects testis from the oxidative damage. Therefore, the testicular glutathione content (both GSH and GSSG levels) was investigated. Our data showed that B(a)P induced decrease in GSH/GSSG ratio (**Figure 6D**) was significantly increased upon curcumin, resveratrol and curcumin-resveratrol co-treatment. These experimental outcomes reflected the ROS scavenging role of curcumin and resveratrol. Moreover, the decrease in the level of GSH is also vulnerable to the activities of GSH related enzymes like GPx. It was also observed that curcumin, resveratrol and curcumin-resveratrol co-treatment with B(a)P significantly increased the activity of GPx in testicular tissue extract (**Figure 6E**). These experimental outcomes proved the protective effect of curcumin and resveratrol against B(a)P mediated oxidative stress.

### Curcumin and Resveratrol Co-treatment Prevents B(a)P Induced Nuclear Translocation of AhR and Subsequent CYP1A1 Expression

**FIGURE 7:**
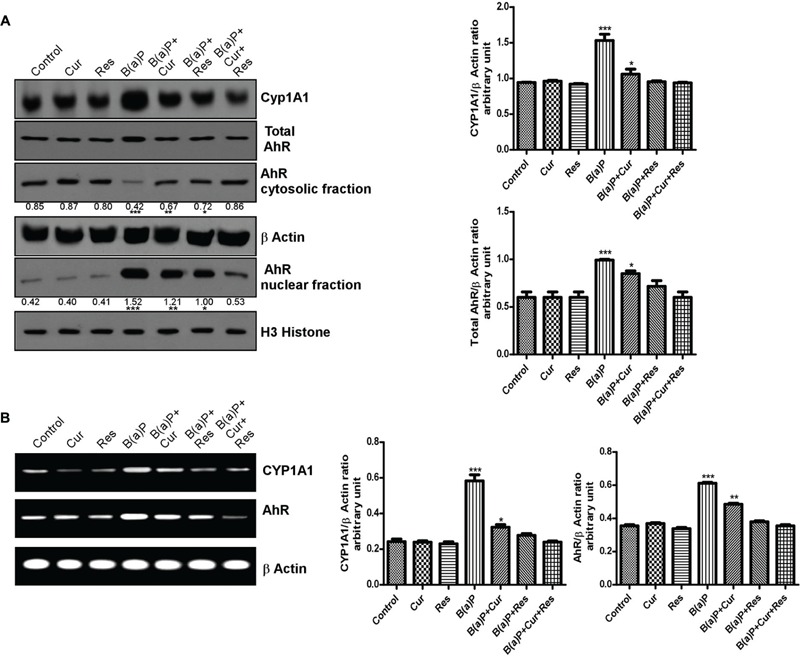
**Curcumin and resveratrol modulate AhR and CYP1A1 expression and protect B(a)P induced testicular germ cell apoptosis. (A)** Western blot of CYP1A1, total AhR, cytosolic and nuclear AhR with isolated testicular germ cells. β Actin was used as the internal loading control for whole cell and cytosolic fractions. Histone H3 was used as the loading control for nuclear fraction. The corresponding histogram (right panel) is the graphical representation of densitometric analysis of CYP1A1 and total AhR, expressing mean ± SEM of relative arbitrary units of the bands for three immunoblots, conducted with separate experiments in each group. One-way ANOVA was followed by Dunnett multiple comparison test. The level of significance was set at (^∗∗∗^*P* < 0.001); (^∗∗^*P* ≤ 0.01–0.001); (^∗^*P* ≤ 0.01–0.05) in respect with the control. **(B)** RT-PCR analysis of CYP1A1 and AhR with isolated testicular germ cells, in different study groups. β Actin was used as the internal loading control. The corresponding histogram (right panel) is the graphical representation of densitometric analysis of CYP1A1 and total AhR. The results were expressed as mean ± SEM. One-way ANOVA was followed by Dunnett multiple comparison test. The level of significance was set at (^∗∗∗^*P* < 0.001); (^∗∗^*P* ≤ 0.01–0.001); (^∗^*P* ≤ 0.01–0.05) as compared with control.

B(a)P is internalized by the cytosolic protein, AhR. Upon ligand binding, AhR translocates to the nucleus (Nebert et al., 1993), where it hetero-dimerises with AhR nuclear translocator (ARNT) protein and binds to the xenobiotic response element (XRE), flanking CYP1A1 gene, thereby activating its transcription. CYP1A1 is the phase I enzyme associated with xenobiotic and drug metabolism. It is involved in the metabolic activation of aromatic hydrocarbons (polycyclic aromatic hydrocarbons) like B(a)P, by transforming it to epoxide. Dietary curcumin has been shown to inhibit B(a)P induced AhR activation, nuclear translocation, DNA binding and subsequent decrease in transcriptional activation of CYP1A1 in different tissue systems (Garg et al., 2008). Resveratrol is also able to block AhR ligand mediated increased initiation of the transcription of CYP1A1 and other phase 1 enzymes both *ex vivo* and *in vivo* (Ciolino et al., 1998). Our previous work (Banerjee et al., 2016) featured about the transcriptional inhibition of CYP1A1 by resveratrol upon B(a)P exposure. Here we investigated the role of curcumin and resveratrol in B(a)P induced AhR expression pattern, nuclear translocation and transcriptional activation of CYP1A1 in testicular germ cells. Our results showed that protein and mRNA expression of CYP1A1 and total AhR were enhanced upon B(a)P treatment (**Figures 7A,B**). Curcumin and resveratrol treatment individually decreased the protein and mRNA expression of CYP1A1 and total AhR. But their combinatorial treatment showed more significant result on CYP1A1 and total AhR expression (**Figures 7A,B**). Curcumin and resveratrol co-treatment significantly decreased nuclear translocation of AhR (**Figure 7A**). This was consistent with the transcriptional activation of CYP1A1. Thus curcumin and resveratrol prevented B(a)P induced nuclear translocation of AhR and decreased CYP1A1 expression (**Figures 7A,B**).

### Curcumin and Resveratrol Co-treatment Prevents B(a)P Induced Activation of Stress Activated Protein Kinases

**FIGURE 8:**
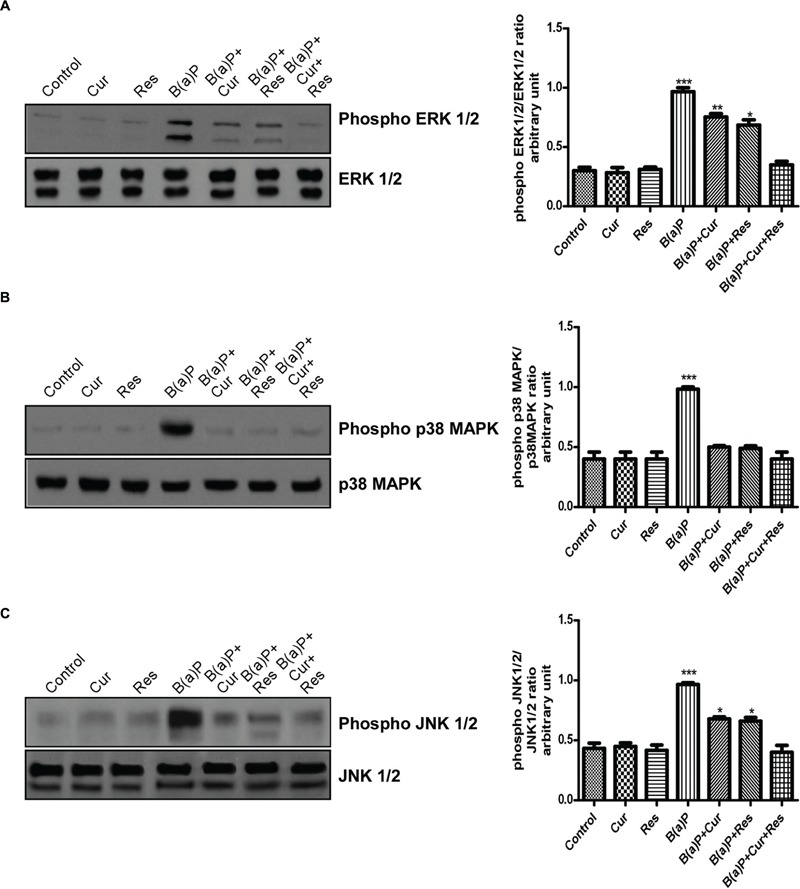
**Curcumin and resveratrol protect testicular germ cells from B(a)P induced apoptosis by modulating MAPK expressions. (A)** Western blot of phospho and total ERK1/2 with isolated testicular germ cells. The corresponding histogram (right panel) represents densitometric analysis of phospho ERK1/2 expression. Histogram is expressing mean ± SEM of relative arbitrary units of the bands for three immunoblots were conducted with separate experiments in each group. One-way ANOVA was followed by Dunnett multiple comparison test. The level of significance was set at (^∗∗∗^*P* < 0.001); (^∗∗^*P* ≤ 0.01–0.001); (^∗^*P* ≤ 0.01–0.05) as compared with control. **(B)** Western blot of phospho and total p38 MAPK. The corresponding histogram (right panel) represents densitometric analysis of phospho p38 MAPK expression. Histogram is expressing mean ± SEM of relative arbitrary units of the bands for three immunoblots were conducted with separate experiments in each group. One-way ANOVA was followed by Dunnett multiple comparison test. The level of significance was set at (^∗∗∗^*P* < 0.001); ^∗∗^(*P* ≤ 0.01–0.001); (^∗^*P* ≤ 0.01–0.05) as compared with control. **(C)** Western blot of phospho and total JNK 1/2. The corresponding histogram (right panel) represents densitometric analysis of phospho JNK 1/2 expression. Histogram is expressing mean ± SEM of relative arbitrary units of the bands for three immunoblots were conducted with separate experiments in each group. One-way ANOVA was followed by Dunnett multiple comparison test. The level of significance was set at (^∗∗∗^*P* < 0.001); (^∗∗^*P* ≤ 0.01–0.001); (^∗^*P* ≤ 0.01-0.05) as compared with control.

The MAPK signaling pathways modulate apoptosis. Our results indicated the involvement of three major MAPKs (ERK1/2, p38 MAPK and JNK1/2) with B(a)P exposure in testicular germ cells. p38 MAPK and JNKs are activated in response to a variety of environmental stresses and inflammatory signals and promote apoptosis and growth inhibition whereas ERK activation is associated with conflicting cellular responses ranging from proliferation and differentiation to apoptosis (Peyssonnaux and Eychene, 2001; Djeu et al., 2002; Lee and McCubrey, 2002). Western blot analysis revealed that B(a)P exposure significantly activated ERK1/2, p38 MAPK and JNK1/2 (**Figure 8**). Resveratrol treatment and curcumin-resveratrol co-treatment significantly decreased the phosphorylation of ERK1/2 (**Figure 8A**). Curcumin and resveratrol treatment and curcumin-resveratrol co-treatment significantly decreased the phosphorylation of p38 MAPK (**Figure 8B**). Curcumin-resveratrol co-treatment significantly decreased the phosphorylation of JNK1/2 (**Figure 8C**).

### Curcumin and Resveratrol Co-treatment Prevents B(a)P Induced Up-regulation and Phosphorylation of p53

**FIGURE 9:**
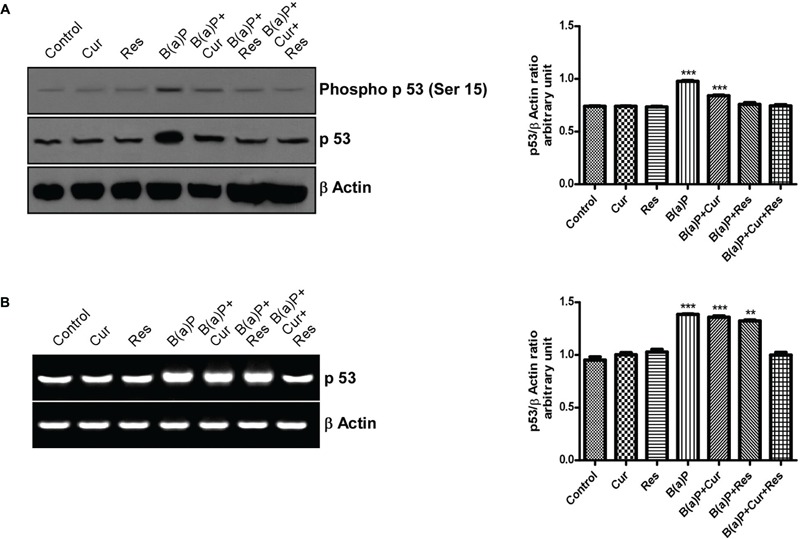
**Involvement of p53 in the B(a)P induced testicular germ cell apoptosis and the protective effect of curcumin and resveratrol. (A)** Western blot of phospho p53 (ser 15) and p53 with isolated testicular germ cells. β Actin was used as the internal loading control. The corresponding histogram (right panel) represents densitometric analysis of phospho p53 (ser 15) expression. Histogram is expressing mean ± SEM of relative arbitrary units of the bands for three immunoblots were conducted with separate experiments in each group. One-way ANOVA was followed by Dunnett multiple comparison test. The level of significance was set at (^∗∗∗^*P* < 0.001); (^∗∗^*P* ≤ 0.01–0.001); (^∗^*P* ≤ 0.01–0.05) in respect with the control. **(B)** RT-PCR analysis of p53 with isolated testicular germ cells, in different study groups. β Actin was used as the internal loading control. The corresponding histogram (right panel) represents densitometric analysis of p53 expression. Histogram is expressing mean ± SEM of relative arbitrary units of the bands for three gels were conducted with separate experiments in each group. One-way ANOVA was followed by Dunnett multiple comparison test. The level of significance was set at (^∗∗∗^*P* < 0.001); (^∗∗^*P* ≤ 0.01–0.001); (^∗^*P* ≤ 0.01–0.05) in comparison with the control.

The p53 is a short-lived, latent transcription factor that act as tumor suppressor to integrate multiple stress signals into a series of diverse anti-proliferative responses. p53 acts as a regulator of the apoptotic process that can modulate key control points in both the extrinsic and intrinsic pathways. Our findings indicated that B(a)P switched on both the mitochondrial and extrinsic apoptotic sequences (Pietsch et al., 2008). Phosphorylation of p53 at Serine 15 (Ser15) is associated with B(a)P induced apoptosis and it is a site targeted by multiple protein kinases (Toledo and Wahl, 2006). Our results showed that B(a)P treatment increased total p53 expression and phosphorylation of p53 at Ser15 in testicular germ cell. Curcumin-resveratrol co-treatment significantly decreased the expression of p53 and phosphorylation of p53 (Ser15) (**Figure 9A**). B(a)P treatment also resulted increased p53 mRNA expression (**Figure 9B**). Curcumin or resveratrol alone was not able to maintain the p53 level upon B(a)P exposure. Curcumin-resveratrol co-treatment significantly decreased the expression of p53 close to the normal (**Figure 9B**).

### Effect of Curcumin on the Pharmacokinetics of Resveratrol and Its Metabolites

Pharmacokinetic study of resveratrol and its phase II metabolites in the presence and absence of curcumin revealed that curcumin treatment enhanced the level of resveratrol and its metabolites in the blood plasma (Supplementary Figure S3; Supplementary Table S1). The Cmax values of resveratrol increased by nearly twofold in the resveratrol-curcumin co-treated condition. The Cmax values of resveratrol glucuronide significantly increased nearly threefolds when animals were treated with curcumin. We did not find any change in Cmax value for resveratrol sulfate. The AUC of resveratrol increased by nearly twofolds in curcumin-resveratrol co-treated animals (Supplementary Table S1). The AUC of resveratrol glucuronide increased nearly twofolds. These findings indicated the better bioavailability of resveratrol in presence of curcumin.

## Discussion

Testicular germ cells consist of spermatogonial cells of different divisional stages, spermatids and mature spermatozoa. Exposure of different environmental toxicants caused damage to the sperm cells, usually the germ line cells. As a result deformed, malfunctioned sperm cells are produced. Our findings indicated that B(a)P induces apoptosis in male reproductive cells particularly the germ cells. Men are easily susceptible to the exposure of B(a)P either through environmental and occupational sources of organic fumes. Different natural phytochemicals could be considered as the potent protective agents against such environmental toxicants. Now a day different natural products are receiving high acceptance as cyto-protective agents because of their high tolerability and low toxicity. Curcumin is a multifunctional natural compound that shows various cyto-protective effects (Aggarwal et al., 2007). Resveratrol being the natural AhR antagonist and antioxidant that has shown promising protective effect against B(a)P induced male reproductive toxicity (Banerjee et al., 2016). The present study was focused on the combinatorial role of curcumin and resveratrol against B(a)P induced male germ cell apoptosis.

Initial findings suggested that curcumin and resveratrol significantly ameliorated B(a)P induced decreased sperm count, motility, circulating testosterone level and prevented testicular degenerative changes. Our focus of study was testicular germ cell populations. That’s why we performed our experiments solely with isolated germ cells of testis. B(a)P exposure resulted significant apoptosis to the germ cell population. Germ cell apoptosis is the major cause for male factor infertility. Curcumin and resveratrol individually were capable to provide partial protection against B(a)P induced germ cell apoptosis. From our dose dependent studies we have selected the single dose for B(a)P, Curcumin and Resveratrol. Our findings indicated that 50 mg curcumin and 50 mg resveratrol individually to some extent prevented germ cell apoptosis at the selected dose of B(a)P (5 mg/kg). But increasing the dose of curcumin or resveratrol did not show any further improvement in their functions. That’s why we were interested to study their combinatorial activity. And our findings surprisingly resulted that the combinatorial dose of curcumin and resveratrol significantly ameliorated B(a)P induced germ cell apoptosis (Supplementary Figure S1).

Oxidative stress is a major cause for germ cell apoptosis (Mishra and Shaha, 2005). Our studies showed that curcumin and resveratrol significantly prevented B(a)P induced testicular ROS generation and oxidative stress. Environmental toxicants induce both intrinsic and extrinsic mode of apoptosis in germ cells (Shaha et al., 2010). Our findings indicated that B(a)P exposure turned on both extrinsic and intrinsic apoptotic pathways. Involvement of intrinsic or mitochondrial pathway is determined by the alteration of cellular Bax/Bcl2 rheostat, cytosolic translocation of mitochondrial cytochrome c and increase in the level of Apaf1. These events eventually activate caspase 9 and 3. Whereas change in Fas/FasL expression and activation of initiator caspase 8 confirm the involvement of extrinsic or death receptor mediated apoptotic pathway. Our findings indicated the simultaneous activation of caspase 9, 8, and 3. These two pathways share molecular crosstalk. As a result both of them got simultaneously activated. In several studies, it has been found that the members of the Bcl2 family of proteins and Fas/FasL system have been implicated in the spermatogenic cell apoptosis under various conditions (Nair and Shaha, 2003; Mishra and Shaha, 2005). Other studies have also focused on the Bcl2 family of proteins as modulators of germ cell survival and death (Kim et al., 2001; Mishra et al., 2006). Our findings suggested that resveratrol was more potent to prevent the onset of mitochondrial apoptotic pathway. Whereas, curcumin and resveratrol co-treatment significantly prevented B(a)P induced mitochondrial as well as death receptor mediated germ cell apoptosis. A recent study reported that curcumin increased the bioavailability of resveratrol and its phase II deposition through inhibiting ABC transporters (Ge et al., 2016). Our findings from pharmacokinetic evaluation indicated that curcumin- resveratrol co-treatment increased the bioavailability of resveratrol and its metabolites in blood. As well as their co-treatment showed more promising result against B(a)P induced cell death in comparison to their individual higher doses (Supplementary Material). So curcumin increased the protective efficiency of resveratrol against B(a)P. Thus the mechanistic study behind curcumin and resveratrol combinatorial treatment came up as the interesting proposal.

p53 is a well-characterized tumor suppressor protein that senses DNA damage, oncogene activation and acts by inhibiting cell cycle progression or by promoting apoptosis. p53 can persuade apoptosis by inducing the transcription of pro-apoptotic members of the Bcl-2 family. These include the ‘multi-domain’ Bcl2 family member Bax (Miyashita et al., 1994), as well as the ‘BH3 only’ members Puma (Nakano and Vousden, 2001), Noxa (Oda et al., 2000) and Bid (Sax and El-Deiry, 2000). p53 can also induce apoptosis by its direct effects on mitochondrial membranes (Olsson et al., 2007). p53 is also known to be involved in the apoptosis of the testis as the spermatogenic cells express p53 mRNA and the protein during apoptosis (Zhu et al., 2000). B(a)P exposure resulted up-regulation of pro-apoptotic genes like Bax, Fas, FasL, Apaf1. p53 acts as the transcription factor for them. We found that curcumin and resveratrol co-treatment significantly attenuated the mRNA expression of the above mentioned genes and thus prevented apoptosis. p53 requires certain post-translational modifications induced by chemicals or stresses. Phosphorylation at serine 15 residue of p53 is an important post-translational modification responsible for the functional efficacy and stability of p53. Several studies have demonstrated the direct association of p53 and its phosphorylation in B(a)P induced apoptosis (Solhaug et al., 2004). Various chemopreventive agents target the phosphorylation status of tumor suppressor genes like p53 (Malhotra et al., 2014). Our experimental results showed that B(a)P exposure significantly increased the p53 protein and mRNA level in germ cells. B(a)P treated group also exhibited significantly increased phosphorylation at ser 15 residue of p53. Curcumin or resveratrol alone did not show significant effect on p53 expression. Supplementation with curcumin and resveratrol in combination brought a significant moderation in the p53 level and its phosphorylation (p53 ser 15) in B(a)P treated germ cells. This decrease in p53 level and its phosphorylation (p53 ser 15) might be one of the prime molecular events utilized by both curcumin and resveratrol to protect the germ cells against B(a)P induced damage.

B(a)P is a PAH that act as an AhR ligand and enters into the cell. Ligand bound AhR acts as the transcriptional activator for CYP1A1 (Tsuji et al., 2014). We observed that B(a)P exposure resulted increase in the total protein and mRNA expression of AhR. B(a)P exposure also induced increased cytosol to nuclear translocation of AhR and increased protein and mRNA expression of CYP1A1. Our results suggested that though curcumin and resveratrol independently was able to inhibit CYP1A1 and AhR expression, but their combinatorial effect was more significant. Thus both curcumin and resveratrol treatment prevented the AhR induced CYP1A1 promoter activation and subsequent modification of B(a)P to its toxic B(a)P-Diol-Epoxide (BPDE) form. This BPDE induces DNA damage and apoptosis. In response to DNA damage, p53 is reported as a central mediator of the cellular responses.

The stress-responsive Mitogen Activated Protein Kinases (MAPKs) have been documented to play a crucial role in regulation of the AhR mediated cellular functions (Tan et al., 2004). MAPKs are stress activated protein kinases comprising ERKs (Extracellular Signal Regulated Kinases), JNKs (c-Jun NH2-terminal Kinases) and p38 MAPKs (Johnson and Lapadat, 2002; Cowan and Storey, 2003; Wada and Penninger, 2004). These Protein kinases are associated with the regulation of cell proliferation, differentiation, stress responses, and apoptosis (Wang et al., 2000; Schweyer et al., 2004; Lu and Xu, 2006; Jia et al., 2009). These three MAPKs are reported to be activated in response to stresses for the induction of apoptosis. MAPKs are activated by phosphorylation at specific residues, then they translocate to the nucleus, where these kinases phosphorylate target transcription factors (Coso et al., 1996; Rosenberger et al., 1999) such as activator protein-1 and p53 (Huang et al., 1999; Toledo and Wahl, 2006). Our results suggested that B(a)P exposure increased phosphorylation of three major MAPKs (ERK 1/2, p38 MAPK, JNK 1/2). Studies have shown that ERKs and p38 kinase physically interact with each other and phosphorylate p53 at serine 15 both *in vivo* and *in vitro* (She et al., 2001). Activated JNK phosphorylates its substrates, c-Jun, ATF2, ELK1, and p53 (Fuchs et al., 1998). Our findings suggested that B(a)P exposure enhanced p53 level and its phosphorylation (Ser 15). We also found that B(a)P treatment increased phosphorylation of ERK 1/2, p38 MAPK and JNK 1/2 in testicular germ cells. That gave us the idea that these MAPKs induced the post-translational modification of p53 for its activation. The treatment with curcumin and resveratrol acted little bit differently from one another. ERK 1/2 activation was significantly inhibited by resveratrol and curcumin-resveratrol co-treatment. Curcumin alone did not show much significant effect on B(a)P induced ERK 1/2 activation. p38 MAPK activation was significantly inhibited by curcumin and resveratrol independent treatment as well as curcumin-resveratrol co-treatment. B(a)P induced JNK 1/2 activation was significantly inhibited only by curcumin-resveratrol co-treatment. These findings gave us the idea that curcumin and resveratrol co-treatment was the suitable protective measure against B(a)P induced MAPK and subsequent p53 activation.

## Conclusion

**FIGURE 10:**
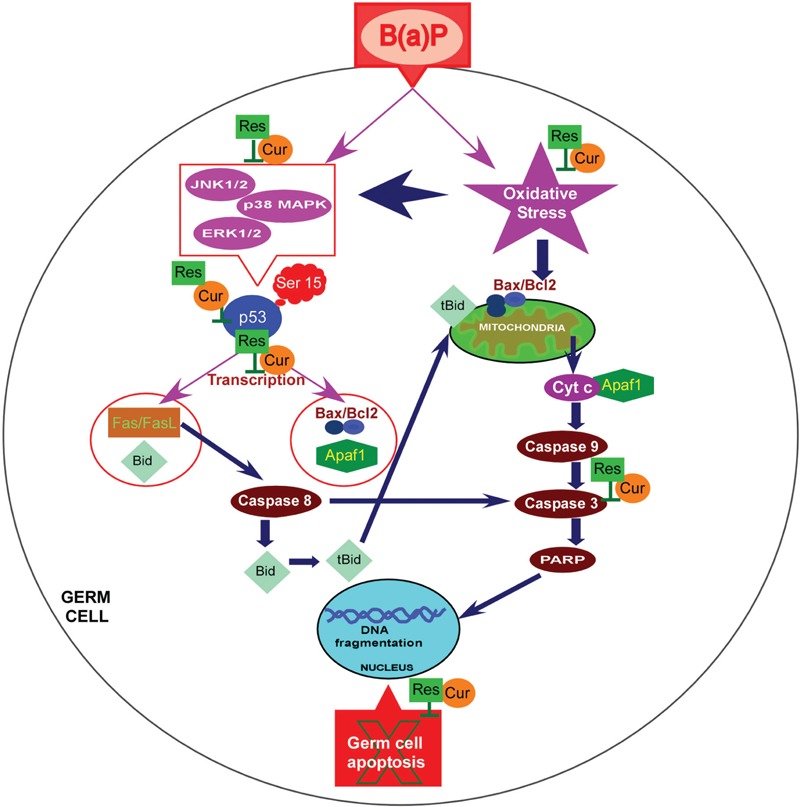
**Schematic representation of the molecular interplay of protective effect of curcumin and resveratrol against B(a)P induced testicular germ cell apoptosis.** B(a)P enters the cell, associates with AhR and induces CYP1A1 promoter activity. B(a)P generates oxidative stress, activates MAPKs (ERK1/2,p38 MAPK,JNK1/2) and p53. B(a)P turns-on intrinsic and extrinsic apoptotic pathway in germ cells. Combinatorial treatment of curcumin and resveratrol prevent CYP1A1 production and quenches oxidative stress. They synergistically attenuate MAPK activation and p53 activation. Curcumin and resveratrol protect testicular germ cells from B(a)P induced apoptosis. B(a)P, Benzo(a)Pyrene; Cur, Curcumin; Res, Resveratrol.

In conclusion, data presented here provide evidence that natural phytochemicals like curcumin and resveratrol effectively prevent B(a)P induced testicular germ cell apoptosis and restore male reproductive health. In the present study we delineated the role of p53 and MAPKs in B(a)P mediated germ cell damage and its protection by curcumin and resveratrol. The study concludes that curcumin increases the efficacy of resveratrol and they synergistically regulate p53 phoshphorylation specifically at ser 15 through the involvement of MAPKs and prevents B(a)P induced apoptosis in testicular germ cells (**Figure 10**).

## Author Contributions

Conceived and designed the experiments: KJ and BB. Performed the experiments: BB and SC. Contributed reagents/materials/analysis tools: KJ and PS. Analyzed the data: KJ and BB. Wrote the paper: BB, KJ, and PS. All authors contributed to and approved the final draft of the manuscript.

## Conflict of Interest Statement

The authors declare that the research was conducted in the absence of any commercial or financial relationships that could be construed as a potential conflict of interest.
