# Supplementary material for: Benzo(a)pyrene Induced p53 Mediated Male Germ Cell Apoptosis: Synergistic Protective Effects of Curcumin and Resveratrol
Source: Front Pharmacol. 2016 Aug 8;7:245. doi: 10.3389/fphar.2016.00245 (PMC4976231; doi:10.3389/fphar.2016.00245)
Supplement: Supplementary file 1 [file Data_Sheet_1.DOCX]

Supplementary Material

**Benzo(a)pyrene induced p53 mediated male germ cell apoptosis: Synergistic protective effects of curcumin and resveratrol**

**Bhaswati Banerjee^1^, Supriya Chakraborty^1^, Debidas Ghosh^2^, Sanghamitra Raha^3^, Parimal C. Sen^1^ & Kuladip Jana^1^***

^1^Division of Molecular Medicine, Bose Institute, Calcutta Improvement Trust Scheme VIIM, Kolkata-700054, India.

^2^Department of Bio-Medical Laboratory Science & Management, Vidyasagar University, Midnapore, West Bengal, India.

^3^ Department of Biotechnology & Integrated Sciences, Visva Bharati, Shantiniketan, Birbhum, West Bengal, India.

***Correspondence:**Dr. Kuladip Jana

[kuladip@jcbose.ac.in/kuladip_jana@yahoo.com](mailto:kuladip@jcbose.ac.in/kuladip_jana@yahoo.com)

**Supplementary Figures**

**
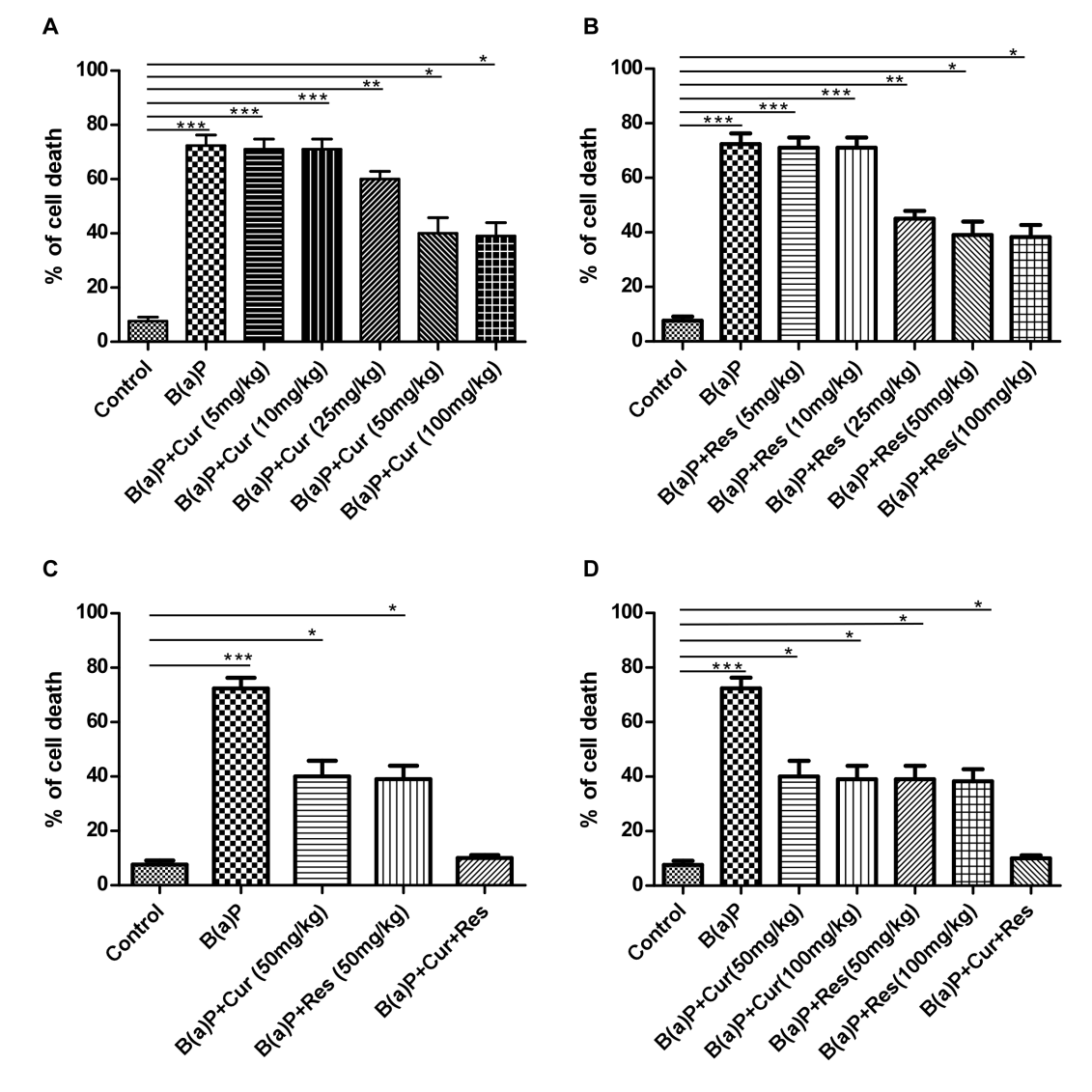
**

**Supplementary Figure 1.** Dose dependent effect of curcumin and resveratrol against B(a)P induced germ cell death. (A) dose dependent effect of curcumin, (B) dose dependent effect of resveratrol, (C) combinatorial effect of curcumin and resveratrol at 50mg/kg (D) combinatorial effect of curcumin and resveratrol at 100mg/kg. The results were expressed as mean ± SEM. One-way ANOVA was followed by Dunnett multiple comparison test. The level of significance was set at ^***^P<0.001; ^**^P ≤0.01-0.001; ^*^P ≤0.01-0.05 in comparison with the control.


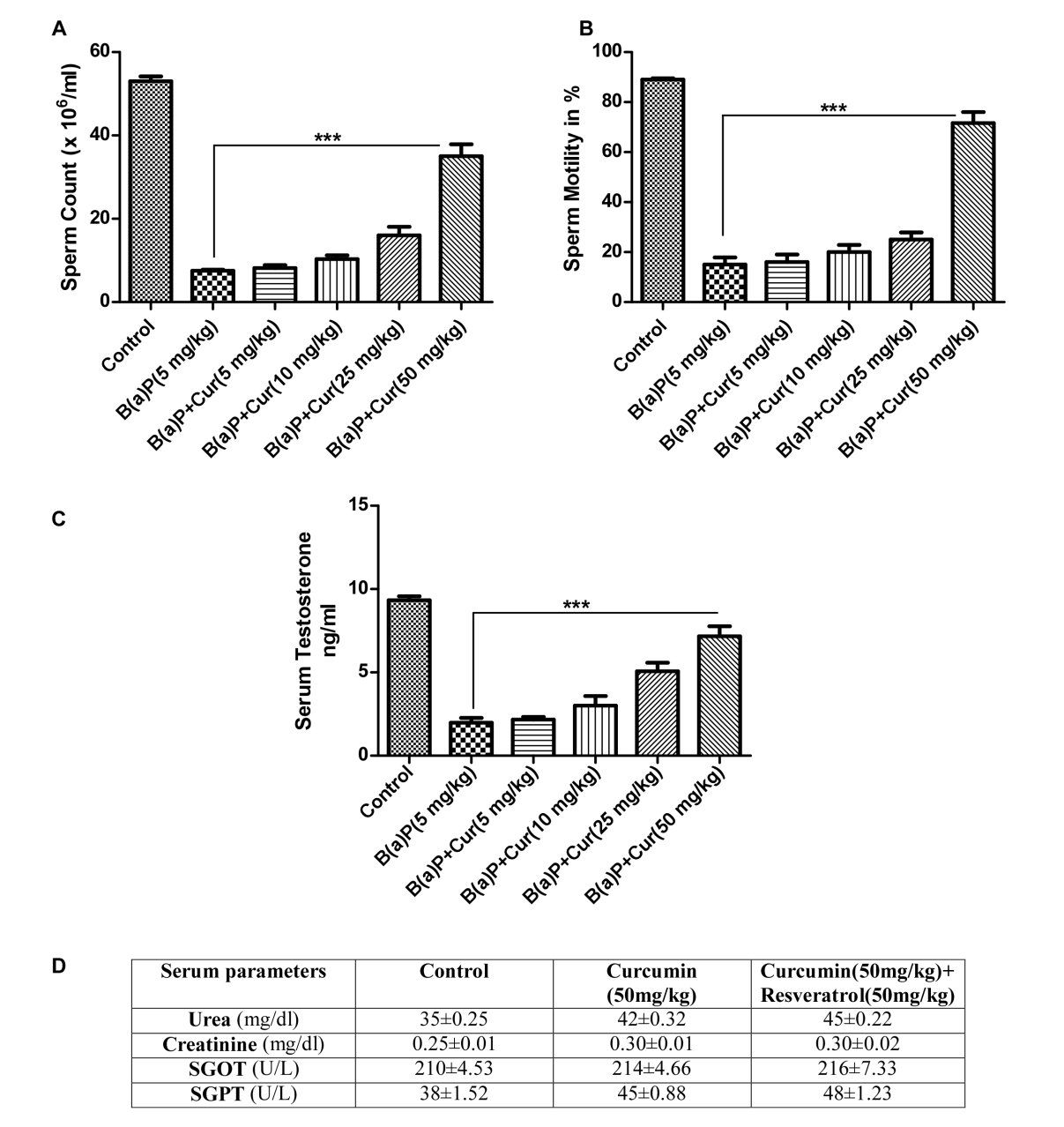


**Supplementary Figure 2.** Dose dependent effect of curcumin against B(a)P induced toxicity. Effect of curcumin on (A) Sperm count, (B) Sperm motility, (C) Serum Testosterone level (D) Effect of curcumin alone and with resveratrol on Serum Urea, Creatinine, SGOT and SGPT. The results were expressed as mean ± SEM. One-way ANOVA was followed by Bonferroni's multiple comparison test. The level of significance was set at ^***^P<0.001;^**^P ≤0.01-0.001; ^*^P ≤0.01-0.05 in comparison with the control.

**
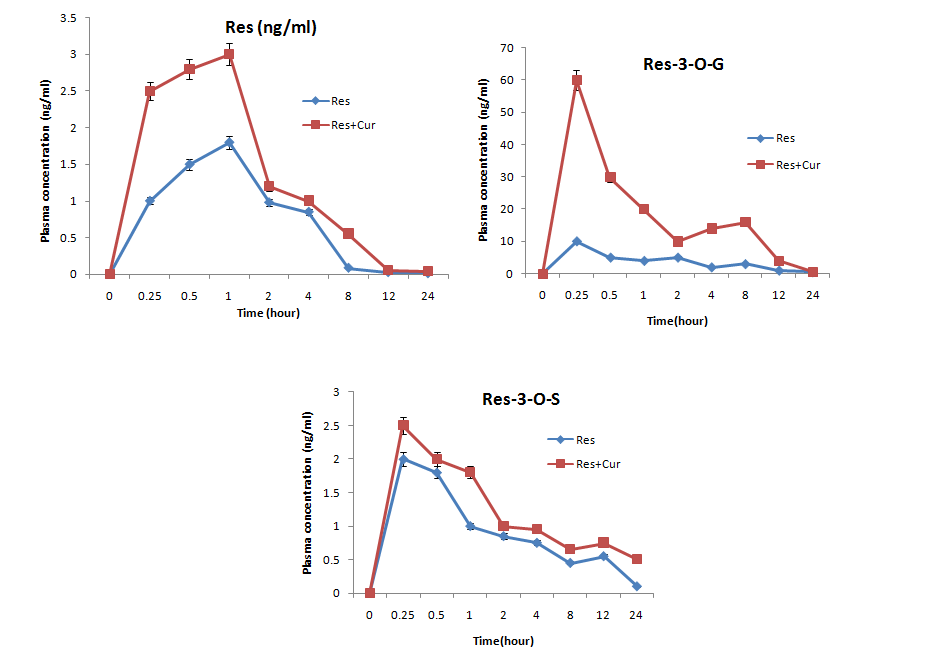
**

**Supplementary Figure 3.** The plasma concentrations of (A) resveratrol (Res), (B) resveratrol-3-O-glucuronide (Res-3-O-G) and (C) resveratrol-3-O-sulfate (Res-3-O-S) after oral administration of resveratrol (50 mg/kg) with or without curcumin (Cur 50 mg/kg) in male wistar rats.

**Supplementary Table 1**

**Pharmacokinetics parameters of resveratrol (Res), resveratrol-3-O-glucuronide (Res-3-O-G) and resveratrol-3-O-sulfate (Res-3-O-S) after oral administration with or without curcumin (Cur).** Values expressed as mean ± SEM.The level of significance was set at ^*^P <0.05.

| **Compounds** | **Treatment groups** | **C_max_(ng/ml)** | **T_max_ (h)** | **AUC_0-t_ (h.ng/ml)** |
| --- | --- | --- | --- | --- |
| **Res** | Res (50mg/kg)  Res (50mg/kg) + Cur (50mg/kg) | 1.20±0.26  2.5±0.54* | 0.43±0.00  0.45±0.00 | 1±0.11  1.967±0.26* |
| **Res-3-O-G** | Res (50mg/kg)  Res (50mg/kg) + Cur (50mg/kg) | 18±5.32  50±6.29* | 0.43±0.15  0.55±0.25* | 40±4.23  70±5.38* |
| **Res-3-O-S** | Res (50mg/kg)  Res (50mg/kg) + Cur (50mg/kg) | 2.32±0.71  2.26±0.52 | 0.45±0.00  0.53±0.24* | 4.5±0.00  4.5±0.12 |
